# Beyond 30% Conversion Efficiency in Silicon Solar Cells: A Numerical Demonstration

**DOI:** 10.1038/s41598-019-48981-w

**Published:** 2019-08-28

**Authors:** Sayak Bhattacharya, Sajeev John

**Affiliations:** Department of Physics, University of Toronto, 60 St. George Street, Toronto, M5S 1A7 Ontario Canada

**Keywords:** Solar cells, Photonic crystals

## Abstract

We demonstrate through precise numerical simulations the possibility of flexible, thin-film solar cells, consisting of crystalline silicon, to achieve power conversion efficiency of 31%. Our optimized photonic crystal architecture consists of a 15 *μm* thick cell patterned with inverted micro-pyramids with lattice spacing comparable to the wavelength of near-infrared light, enabling strong wave-interference based light trapping and absorption. Unlike previous photonic crystal designs, photogenerated charge carrier flow is guided to a grid of interdigitated back contacts with optimized geometry to minimize Auger recombination losses due to lateral current flow. Front and back surface fields provided by optimized Gaussian doping profiles are shown to play a vital role in enhancing surface passivation. We carefully delineate the drop in power conversion efficiency when surface recombination velocities exceed 100 *cm/s* and the doping profiles deviate from prescribed values. These results are obtained by exact numerical simulation of Maxwell’s wave equations for light propagation throughout the cell architecture and a state-of-the-art model for charge carrier transport and Auger recombination.

## Introduction

Photovoltaics provides a very clean, reliable and limitless means for meeting the ever-increasing global energy demand. Silicon solar cells have been the dominant driving force in photovoltaic technology for the past several decades due to the relative abundance and environmentally friendly nature of silicon. Nevertheless, one of the drawbacks of crystalline silicon is the indirect nature of its electronic band gap, making it a relatively weak absorber of long wavelength sunlight. Traditionally, this has been offset using a relatively thick (100–500 *μm*) silicon structure. While enabling more solar absorption, thicker silicon adds to the materials cost for large area applications and renders the structure inflexible. Moreover, thick silicon solar cells suffer from unavoidable losses in power conversion efficiency due to non-radiative recombination of photo-generated charge carriers during their relatively long path to electrical contacts at the extremities of the cell. These deficiencies have sparked broad interest in a variety of thin-film solar materials including *CdTe*, *GaAs*, perovskites and various polymers^1–3^. Due to the indirect band gap nature of *c*–*Si*, thin-film silicon has not been considered a viable competitor to these alternative materials.

In some recent papers^4,5^, we have suggested a paradigm shift in solar science and technology, exploiting the wave nature of sunlight while retaining a realistic description of charge-carrier recombination. By designing suitable photonic crystal architectures that promote wave-interference based light-trapping in the required frequency band, it is possible for *c*–*Si* thin films to absorb sunlight as effectively as a direct band gap semiconductor. In this paper we demonstrate how this enables a flexible, 15 *μm*-thick *c*–*Si* film with optimized doping profile, surface passivation and interdigitated back contacts (IBC) to achieve a power conversion efficiency of 31%, higher than that of any other single material of any thickness.

The maximum possible room-temperature power conversion efficiency of a single junction, *c*–*Si* solar cell under 1–sun illumination, according to the laws of thermodynamics, is 32.33%^6^. This limit is based on the assumptions of perfect solar absorption and no losses due to non-radiative charge-carrier recombination. The best real-world silicon solar cell to date, developed by Kaneka Corporation, is able to achieve 26.7% conversion efficiency^7,8^. A loss analysis of this 165 *μm*-thick, heterojunction IBC cell shows that in absence of any extrinsic loss mechanism the limiting efficiency of such a cell would be 29.1%^7^. The competing factors responsible for this upper limit of the conversion efficiency are ray-optics based light-trapping and intrinsic loss due to Auger charge-carrier recombination^9,10^. The thicker the cell, the more light is absorbed. Unfortunately, this is accompanied by increased bulk non-radiative recombination loss of charge-carriers. In the hypothetical case of ideal Lambertian light-trapping, state-of-the-art Auger charge-carrier recombination^11^ and the inclusion of band gap narrowing (BGN) in *c*–*Si*, a theoretical limit to power conversion efficiency of 29.43% has been proposed^10^. In this case, the optimum balance between solar absorption and bulk losses is achieved for a cell of 110 *μm* thickness. In traditional light trapping structures, the Lambertian limit is not achieved and the optimum solar cell thickness is much greater than 110 *μm*, as witnessed by the world-record-holding Kaneka cell. Moreover, the inclusion of non-zero bulk doping and surface charge carrier recombination effects further reduce the theoretical power conversion limit by at least another (additive) percentage point. For these reasons, light-trapping concepts using ray-optics, applied to any conventional silicon solar cell architecture, are not expected to yield power conversion efficiencies beyond 28%.

The wave nature of light offers a powerful alternative paradigm for solar energy capture and conversion in silicon. This is evident in certain sub-wavelength scale waveguides^12–14^ and photonic crystal^15,16^ architectures with microstructure periodicity and feature sizes on the scale of near-infrared light^17–22^. Sunlight that would otherwise be weakly absorbed in a thin film is, instead, absorbed almost completely. The resulting photonic crystal solar cell absorbs sunlight well beyond the longstanding Lambertian limit. This, in turn, leads to a dramatic reduction in the optimum silicon solar cell thickness. Ray-optics is an approximation that cannot be applied to photonic crystals and accurate modeling of wave-interference based light-trapping in a photonic crystal (PhC) due to multiple coherent scatterings from wavelength-scale micro-structures requires rigorous numerical solution of Maxwell’s equations^17–23^ throughout the solar cell architecture. A coupled optical-electronic approach and experimental study on a 3 *μm*-thick cell in^23^ showed the possibility of enhanced light-absorption and conversion efficiency in patterned silicon cells as compared to bare silicon cells. However, the light-absorption in this study still falls well below the Lambertian light-trapping limit.

Recent coupled optical-electronic analysis of thin-silicon solar cells involving parabolic pore PhCs^4^ and inverted pyramid PhCs^5^ have shown that the previous theoretical efficiency limit obtained by ray-optics based Lambertian light-trapping can be surpassed. In contrast to 165 *μm*-thick Kaneka cell and 110 *μm*-thick optimum Lambertian cell, photonic crystal solar cells are an order of magnitude thinner. The key mechanisms enabling nearly 30% efficiency using just 10 *μm*-thick silicon are existence of long-lifetime, slow-light resonances, parallel-to-interface refraction (PIR) and the coupling into such modes from external plane waves^24^. Slow-light modes exhibiting vorticity in the Poynting vector flow originate from wave-interference and cannot be achieved by ray-optics based Lambertian light-trapping. They require silicon microstructures on the scale of the optical wavelength. The Lambertian limit involves a number of assumptions such as, a randomly rough top surface without any specular reflection and deflection of the incident rays according to a cos*θ* probability distribution, where *θ* is the angle between the rays inside the slab and the surface normal. According to this model, parallel to interface flow of light (i.e. deflection of light rays at nearly *θ* = 90°) is unattainable. Light waves in PhCs exhibit behavior beyond the realm of ray-optics with the potential to bridge the gap between the thermodynamic efficiency limit and ray-optics based limits. Although thin-silicon PhC solar cell designs with front contacts, discussed earlier^4,5^, are capable of achieving efficiencies up to 30%, optical shadowing loss due to front contacts and power loss due to sheet resistance prevent them from substantially surpassing this limit.

In this article, we demonstrate that thin-silicon PhC solar cells with IBC can surpass the 30% power conversion efficiency barrier. We consider 3–20 *μm* thick, flexible *c*–*Si* IBC cells with a *p*-type bulk doping concentration of 5 × 10^15^ *cm*^−3^. These inverted micro-pyramid photonic crystals are optimized for light-trapping using an exact finite difference time domain (FDTD) simulation of Maxwell’s equations throughout the cell for each cell-thickness. The optical generation profiles for the optimized PhCs are then used for carrier transport optimization. We show that each optimized silicon PhC is capable of achieving a photo-current density well beyond Lambertian limit. We also present a physical explanation for the underlying wave-interference mechanism responsible for this unprecedented light trapping and absorption capability. The PhC solar cells exhibit multiple resonant peaks in the 900–1200 *nm* wavelength range of the absorption spectra, a region where conventional silicon solar cells and planar cells absorb negligible sunlight. These resonant peaks of PhCs are associated with PIR and vortex like flow of trapped solar energy that gives rise to effective path lengths much longer than the 4*n*^2^ path-enhancement associated with Lambertian limit. Our electronic optimization of the IBC cell involves realistic Gaussian doping profiles of emitter, back surface field (BSF) and front surface field (FSF) regions. We optimize contact geometry and widths through careful consideration of BGN, Auger recombination and practically feasible Shockley-Read-Hall (SRH) lifetimes. As the cell-thickness increases, the short-circuit current of the cell increases due to more light-absorbing material. As expected, increased cell-thickness reduces the open-circuit voltage of the cell due to increased bulk-recombination, leading to a new optimum IBC cell-thickness. This balance between light-absorption and bulk recombination suggests an optimum thickness slightly larger than that of the corresponding front contact solar cell^5^. We consider a wide range of SRH lifetime and study the effect of lifetime variation on optimum cell-thickness. Our results suggest that for SRH lifetimes exceeding 1 *ms*, the optimum PhC IBC cell-thickness is 15 *μm*, in contrast to 110 *μm* optimum thickness of the hypothetical Lambertian cell. For SRH lifetimes 1 *ms* and 10 *ms* and contact SRV 10 *cm*/*s*, our optimum 15 *μm* PhC IBC cell yields power conversion efficiencies of 30.29% and 31.07%, respectively. Even when the contact SRV increases to 100 *cm*/*s*, our optimum cell delivers close to 31% conversion efficiency. Our thin-film photonic crystal design provides a recipe for single junction, *c*–*Si* IBC cells with ~4.3% more (additive) conversion efficiency than the present world-record holding cell using an order of magnitude less silicon.

Ray-trapping architectures in traditional silicon solar cells usually employ two types of surface textures: upright and inverted pyramids^25–31^. Randomly distributed upright pyramid textures are widely used due to their easy mask-less fabrication through *KOH* etching of the silicon surface. Despite easy fabrication, upright-pyramid, thin-silicon structures typically provide less effective light-trapping than the optimized inverted-pyramid PhC of the same thickness^32^. On the other hand, a regular array of inverted pyramids has been used for light-trapping in the previous record-holding, passivated-emitter, rear locally diffused (PERL) cell with 25% conversion efficiency and 400 *μm*-thickness^31^. However, the feature-sizes of traditional inverted pyramid cells are typically 10 *μm* or more and light-absorption in such cells falls below the Lambertian ray-trapping limit. Traditional ray-trapping architectures require thick silicon (~160–400 *μm*) to achieve sufficient light absorption, with concomitant bulk carrier recombination that usually limits the conversion efficiency to below 27%. In contrast, our light-trapping geometry employs inverted pyramids with base-lengths ranging between 1.3–3.1 *μm*. This allows our cells to achieve beyond-Lambertian light-absorption through strong wave-interference effects. Using only 3–20 *μm*-thick silicon, resulting in low bulk-recombination loss, our silicon solar cells are projected to achieve up to 31% conversion efficiency, using realistic values of surface recombination, Auger recombination and overall carrier lifetime.

Although the surface of our silicon solar cell is patterned along a plane that is perpendicular to the incident light, the light-propagation characteristics are considerably richer than widely-studied grating-coupled waveguides. Our photonic crystal refracts and diffracts incoming light to numerous wave-vectors that are nearly parallel to the air-silicon interface. These wave-vectors couple to and experience the long-lifetime slow-light modes of the PhC. Vortex-like flow of the electromagnetic Poynting vector is evident in high density of optical resonances throughout the 800–1200 *nm* range. These modes are evidence of an enhancement of the overall electromagnetic density of states over this wavelength range and are characteristic of the higher bands of a photonic crystal. In contrast, the grating couplers exhibit a much narrower coupling band-width, typically about 10% of center frequency^33–37^.

## Solar cell Geometry and Numerical Details

**Figure 1:**
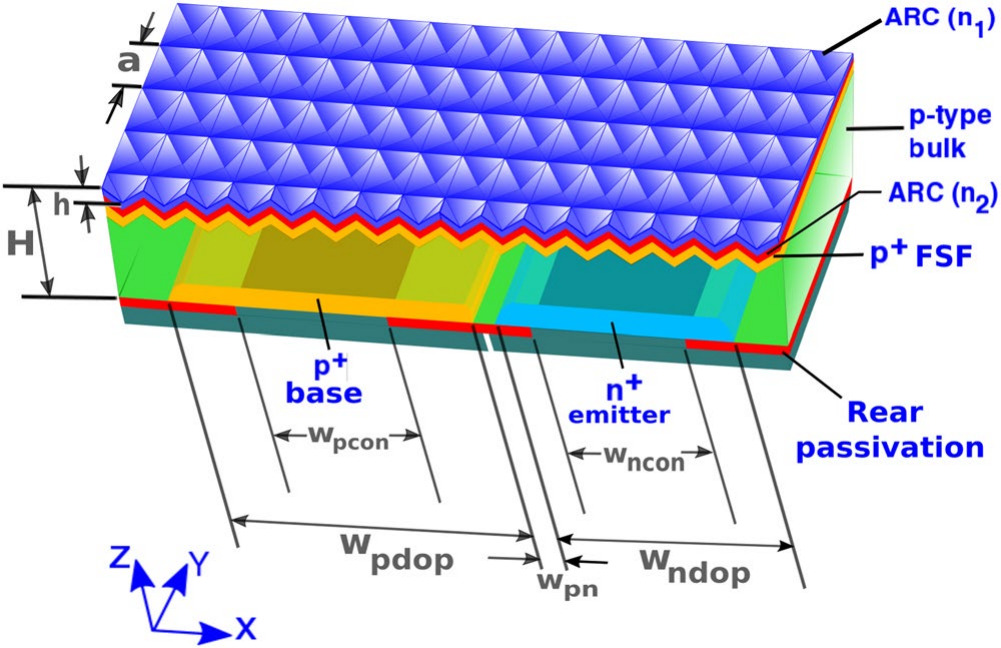
Geometry of the proposed inverted-pyramid photonic crystal IBC solar cell. The front surface of the cell is textured with a square lattice of inverted pyramids and coated with dual-layer ARC with refractive indices *n*_1_ and *n*_2_. The thickness of the silicon layer is given by *H*.The *p*-type bulk has a uniform doping concentration of 5 × 10^15^ *cm*^−3^. *w*_*pdop*_ and *w*_*ndop*_ denote the widths of the base and emitter dopings, respectively. The base and emitter contact widths are denoted by *w*_*pcon*_ and *w*_*ncon*_, respectively. *w*_*pn*_ represents the distance between the edges of the base and emitter regions of the cell.

Figure 1 shows the schematic of our PhC-IBC cell. The front surface of the solar cell is textured with a square lattice of inverted micro-pyramids of lattice constant *a*. Such inverted pyramids are fabricated by *KOH* etching of the (100) surface of silicon, exposing the (111) surfaces and resulting in a pyramid side-wall angle of 54.7°^21^. The cell has a dual-layer antireflection coating (ARC) of refractive indices *n*_1_ and *n*_2_ and thicknesses *t*_1_ and *t*_2_, respectively. This ARC layer also acts as part of the front passivation of the cell. We consider *c*–*Si* cells with thickness (*H*) ranging over 3–20 *μm*. The *p*-type bulk is assumed to have a uniform doping concentration of 5 × 10^15^ *cm*^−3^. Both the front surface field (FSF) and the base consist of highly doped *p*-regions (denoted by *p*^+^) with Gaussian doping profiles. Similarly, the emitter contains a highly *n*-doped region (*n*^+^) with Gaussian doping profile. The peak doping concentrations of *n*^+^ and *p*^+^ regions are denoted by *N*_*n*0_ and *N*_*p*0_, respectively. The corresponding Gaussian doping profiles are ${N}_{i0}\exp (-\,{z}^{2}/2{\sigma }_{i}^{2})$, where *i* = *n*, *p*. Here, *z* refers to the direction of the Gaussian variation and *σ*_*i*_ denotes the depth of the doping profile. The widths of the base and emitter regions are assumed to be *w*_*pdop*_ and *w*_*ndop*_. The separation between the edges of emitter and base is *w*_*pn*_. A rear passivation layer covers all back surfaces of the cell where the electrode fingers do not make direct contact with the *n*^+^ and *p*^+^ regions. The width of the base (emitter) contact, touching the *p*^+^ (*n*^+^) region, is denoted by *w*_*pcon*_ (*w*_*ncon*_). The emitter and base contacts extend below the rear passivation and acts as back-reflector for the cell.

A stable FDTD scheme, implemented using open source software package Electromagnetic Template Library (EMTL)^38^, is used to simulate Maxwell’s equations and optimize the light-trapping performance of the *c*–*Si* solar cell. A unit cell of the *c*–*Si* inverted pyramid PhC is used for 3*D* FDTD computations. Perfectly matched layers (PML) are applied at the top and bottom boundary planes (normal to *z*-direction) of the computation domain. Periodic boundary conditions are assumed along the *x* and *y*− directions. The top of the inverted pyramids are coated with dual ARC layers. The bottom of the *c*–*Si* is coated with a 50 *nm SiO*_2_ passivation layer (with refractive index 1.45), backed by a perfect electric conductor (PEC), acting as a back-reflector to the sunlight. The use of *SiO*_2_ buffer layer reduces parasitic absorption losses in real-world back contact such as silver^39^ and justifies the use of a PEC to simulate the back-reflector. Calculation of absorbed photon density in our PhC solar cell is a two-step process. In both steps, the cell is illuminated with a broadband plane wave, incident from +*z*-direction. In the first step, the incident wave has significant energy in the 300–1100 *nm* spectral range. In the second step, we accurately model solar absorption in the 1100–1200 *nm* range. This latter absorption in *c*–*Si* involves both electronic bandgap narrowing (BGN)^40^ and phonon-assisted optical absorption comprising the Urbach edge^41–44^. As we show in sec. 3, the second effect is insignificant in conventional Lambertian light-trapping based solar cells but contributes significant sub-gap solar absorption in our PhC solar cell. In the second FDTD computation, we use an incident plane wave with significant energy in the 1100–1200 *nm* spectral range. A detailed model of the complex refractive index of *c*–*Si* in the 1100–1200 *nm* wavelength range appears in the “Methods” section.

Combining the results of the separate FDTD computations, we calculate the absorption coefficient of the *c*–*Si* over 300–1200 *nm* wavelength range as *A*(*λ*) = 1 − *R*(*λ*) − *T*(*λ*), where *R*(*λ*) and *T*(*λ*) are the reflection and transmission coefficients of the structure. The maximum achievable photo current density (MAPD) of the cell under *AM*1.5*G* illumination is given by:

1 $${J}_{MAPD}={\int }_{\lambda =300\,nm\,}^{\lambda =1200nm}\frac{e\lambda }{hc}I(\lambda )A(\lambda )d\lambda $$

Here, *I*(*λ*) is the intensity of the *AM*1.5*G* spectrum. We assume that each absorbed photon creates a single electron-hole pair. The short-circuit current (*J*_*SC*_) of an ideal cell, without any surface and bulk recombination losses, coincides with *J*_*MAPD*_.

**Figure 2:**
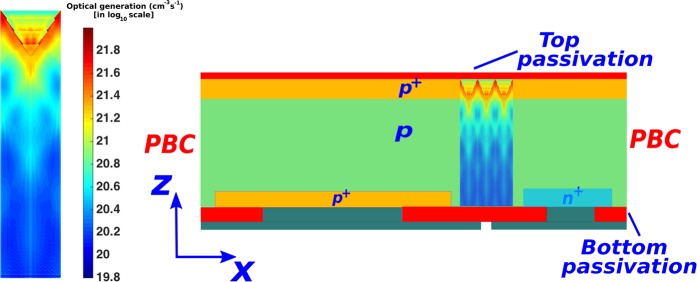
*2D* transport model of the inverted pyramid photonic crystal IBC solar cell shown in Fig. 1. The optical generation, shown on the left side, is calculated using 3*D* FDTD computation that involves a unit cell of the inverted pyramid photonic crystal. This 3*D* generation profile is then integrated over *y*-direction and repeated over multiple photonic crystal unit cells to cover the entire width of the IBC cell. For illustration purpose, we have only shown the optical generation in a 10 *μm*-thick cell but each cell-thickness under consideration involves its own optical generation profile. Periodic boundary condition (PBC) is used along *x*-direction in our transport calculations.

The left panel of Fig. 2 shows a sample optical generation profile for a 10 *μm*-thick cell obtained through our FDTD calculation. The actual 3*D* profile has been integrated along the *y*-direction and converted into an equivalent 2*D* profile. This 2*D* profile is then repeated over many unit cells of the inverted pyramid PhC to cover the entire width of the 2*D* transport model of the IBC cell (shown in the right panel of Fig. 2). For the purpose of clarity, the carrier generation profile only over 3 unit cells is shown. The 2*D* carrier transport calculations are performed using Sentaurus^45^ assuming a temperature of 25 °*C*. In all the calculations, the Shockley-Read-Hall (SRH) lifetime, *τ*_*SRH*_, is assumed to be 10 *ms* (except for the cases where we study the performance and optimum thicknesses of our solar cell as a function of *τ*_*SRH*_) according to the experimental results obtained in^11^. The Auger recombination in our carrier-transport calculations is implemented using the state-of-the-art improved Auger model^11^. The surface recombination at the *Si* − *SiO*_2_ interface is implemented using a microscopic, SRH recombination statistics-based model^46^ (more details are given in the “Methods” section) that complies with the experimental data of^11,47–49^. In all our computations involving inverted-pyramid PhC solar cells, the contact SRVs are chosen to be 10 *cm*/*s*. This low contact SRV allows us to compare the performance of our solar cell to the benchmarks that completely neglect surface recombination^10^. In addition, recent experimental developments suggest that passivated contacts allow realization of IBC cell with much lower contact SRVs than conventional contacts^50^. Nevertheless, we discuss the effect of higher contact SRVs on the performance of our cell in sec. 4.

One approach to achieve low SRVs is through passivated carrier-selective contacts using highly doped polycrystalline *Si* (poly-*Si*) thin films. Experimental study^51^ has shown that a 20 *nm*-thick poly-*Si* front-contact leads to parasitic absorption loss of ~1.1 *mA*/*cm*^2^. In contrast, in our IBC design, poly-*Si* layers with similar thickness would be placed at the back of the cell. As shown by the integrated optical generation profile of Fig. 2, our photonic crystal architecture captures the vast majority of sunlight in the upper parts of the cell. Two orders of magnitude more photogenerated carrier density appears in the upper regions relative to the bottom of the cell. Accordingly, we expect that parasitic absorption in poly-*Si* bottom contacts is negligible in our PhC-IBC cell.

For all our charge-carrier transport calculations, we assume that the the contacts are ideal (i.e. zero resistivity). The resistive losses in the bulk and along the highly doped *n*^+^ and *p*^+^ regions depend on the doping concentrations and are automatically considered in our 2*D* drift-diffusion calculations. We account for the losses at the semiconductor-dielectric and semiconductor-metal interfaces through SRH recombination statistics^46^ and contact surface recombination velocities, respectively.

## Light-Trapping Optimization

**Figure 3:**
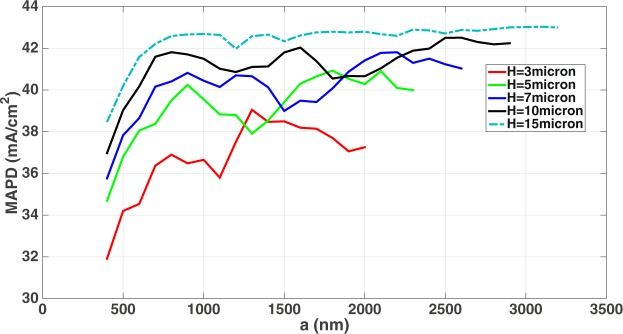
Light-trapping optimization in the 300–1100 *nm* wavelength range for inverted pyramid *c*–*Si* photonic crystals with thickness *H* and dual-layer ARCs with *n*_1_ = 1.4, *t*_1_ = 45 *nm*, *n*_2_ = 2.6 and *t*_2_ = 100 *nm*. Here, *a* denotes the lattice constant of the photonic crystal. The details of the optimum lattice sizes are summarized in Table 1.

*C*–*Si* thin-films with low doping can provide solar cells with high open-circuit voltage due to reduced bulk recombination, but usually suffer from poor solar absorption. Maximization of light-trapping capability in *c*–*Si* thin-film is one of the most important aspects of high-efficiency, ultra-thin silicon solar cell design. We show below that 3–20 *μm*-thick *c*–*Si* inverted micro-pyramid PhCs are highly effective for wave-interference based light-trapping leading to solar absorption, comparable to (and in some cases more than) that of the 165–400 *μm*-thick conventional cells. Our simulations reveal that a dual-layer ARC with *n*_1_ = 1.4, *t*_1_ = 45 *nm*, *n*_2_ = 2.6 and *t*_2_ = 100 *nm* exhibits the best anti-reflection behavior, irrespective of the cell-thickness. Figure 3 shows the optimization results for the lattice constant, *a*, of *c*–*Si* inverted-pyramid PhCs with *H* = 3, 5, 7, 10, 15 *μm* for MAPDs over 300–1100 *nm* wavelength range. We also note from Fig. 3 that for a 3 *μm*-thick cell, the MAPD corresponding to *a* = 400 *nm* is 31.9 *mA*/*cm*^2^ in comparison to the MAPD of 39.05 *mA*/*cm*^2^ at the optimum lattice constant of 1300 *nm*. However, as the cell becomes thicker, this difference progressively decreases. For a 20 *μm*-thick cell, the difference between MAPD at *a* = 2900 *nm* and *a* = 400 *nm* is only 0.34 *mA*/*cm*^2^. For *H* = 15 *μm*, the MAPD shows a maximum variation of 0.25 *mA*/*cm*^2^ over the 1700–3200 *nm* lattice constant range. The light-trapping performances of 15–20 *μm*-thick inverted PhC solar cells are extremely robust with respect to lattice constant variation. The total MAPD over the entire 300–1200 *nm* wavelength range, for the optimum cases of different cell-thickness, are shown in Table 1. The 1100–1200 *nm* absorption is calculated according to an accurate Lorentz model of experimental *c*–*Si* dispersion data as discussed in the “Methods” section. Table 1 also shows that wave-interference based light-trapping in our optimized thin-silicon inverted pyramid PhCs surpasses the ray-optics based Lambertian light-trapping limit. The MAPDs corresponding to the Lambertian limits of different cell-thicknesses are calculated using absorption coefficient from^9^ and eq. 1.

**Table 1:** Summary of wave-interference based light-trapping optimization in 3–20 *μm*-thick inverted pyramid PhC solar cells.

*H*(*μm*)	*a*(*nm*)	MAPD corresponding to Lambertian limit (*mA*/*cm*^2^), 300–1200 *nm* range	MAPD of inverted pyramid PhC solar cell (*mA*/*cm*^2^), 300–1100 *nm* range	MAPD of inverted pyramid PhC solar cell (*mA*/*cm*^2^), 1100–1200 *nm* range	Total MAPD of inverted pyramid PhC solar cell (*mA*/*cm*^2^), 300–1200 *nm* range
3	1300	36.64	39.05	0.31	39.36
5	1800	38.03	40.93	0.63	41.56
7	2100	38.85	41.81	0.98	42.79
10	2500	39.63	42.50	1.09	43.59
12	2700	40.01	42.75	1.24	43.99
15	3100	40.44	43.03	1.36	44.39
18	1900	40.78	43.11	1.34	44.45
20	2900	40.97	43.12	1.39	44.51

The inverted-pyramid PhC solar cells are assumed to have dual-layer ARCs with *n*_1_ = 1.4, *t*_1_ = 45 *nm*, *n*_2_ = 2.6 and *t*_2_ = 100 *nm*. Each of our inverted pyramid photonic crystals, optimized through stable and accurate solutions of Maxwell’s equations, has MAPD considerably above the Lambertian limit.

**Figure 4:**
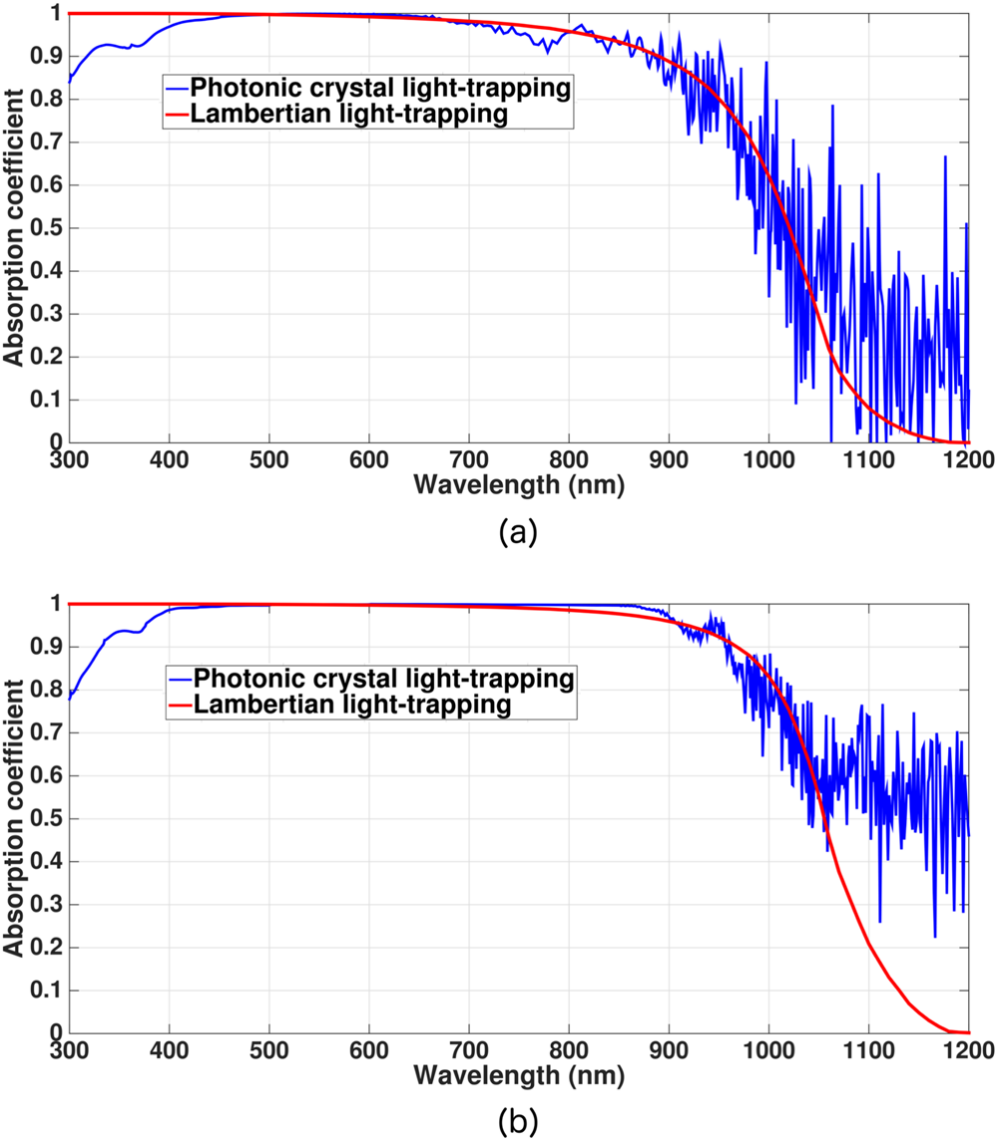
Absorption spectra for (**a**) 5 *μm* and (**b**) 15 *μm*-thick inverted pyramid photonic crystal IBC solar cell over the 300–1200 *nm* wavelength range (blue curves). The 1100–1200 *nm* absorption is modeled following the details illustrated in the “Methods” section. Red curves correspond to the absorption spectra of the hypothetical Lambertian cells. In contrast to Lambertian cells and planar cells, high solar energy absorption in the 950–1200 *nm* spectral range due to multiple resonant absorption peaks is a signature of photonic crystal light-trapping.

**Figure 5:**
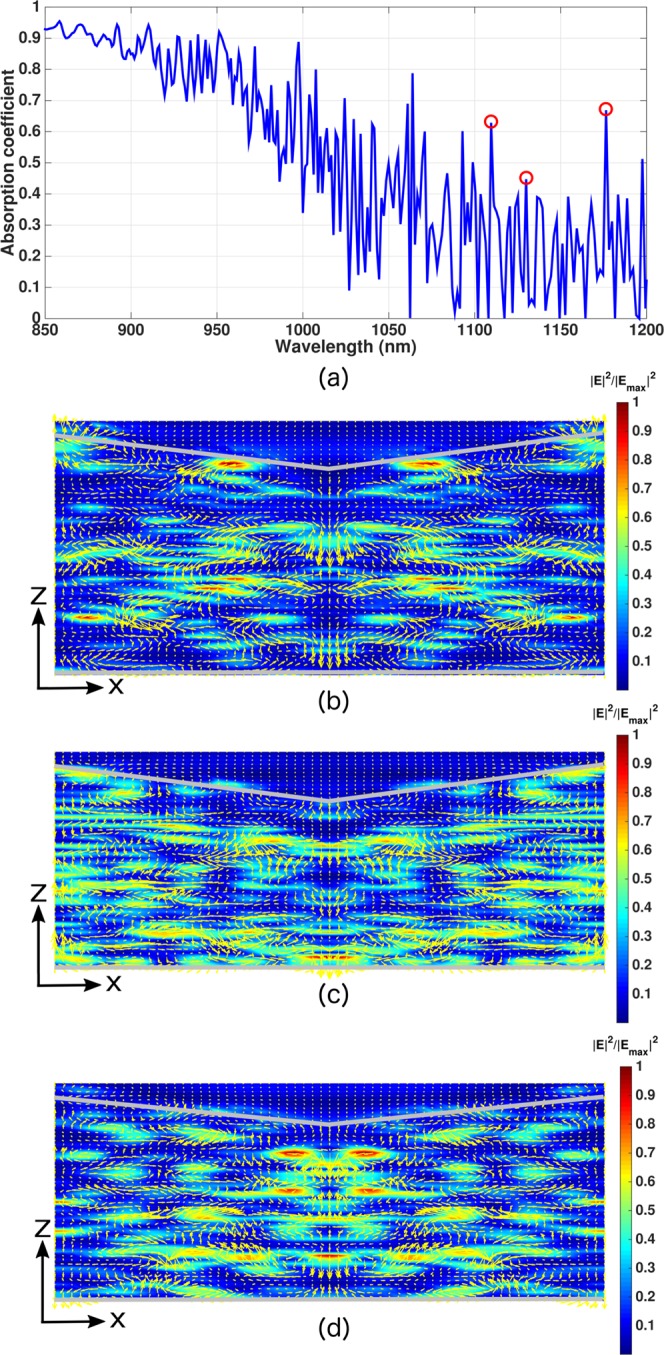
Parallel-to-interface refraction (PIR) and vortex-like modes in inverted pyramid photonic crystal with *H* = 5 *μm* and *a* = 1800 *nm*: (**a**) Magnified absorption spectrum of the cell over the 850–1200 *nm* wavelength range. The red circles correspond to the resonances at *λ* = 1110,1130 and 1176 *nm*. (**b**), (**c**) and (**d**) Yellow arrows show the in-plane Poynting vector flow over the central *xz*-plane of the inverted pyramid PhC unit cell at *λ* = 1110, 1130 and 1176 *nm*, respectively. Such long lifetime modes are responsible for the high absorption even in the 1100–1200 *nm* wavelength range, in sharp contrast to Lambertian and planar cells.

As illustrative examples of our optimized inverted pyramid PhC solar cells, we show two absorption spectra in Fig. 4 over the 300–1200 *nm* wavelength range: a thin cell with *H* = 5 *μm* and a relatively thicker cell with *H* = 15 *μm*. These absorption spectra exhibit multiple resonance peaks and significant absorption in the 900–1200 *nm* wavelength range, whereas Lambertian cells and planar silicon are weak absorbers of sunlight. These peaks in the absorption spectra originate from purely wave-interference effects, absent in Lambertian light-trapping. To illustrate this point, we show a magnified view of the absorption spectrum of the 5 *μm*-thick, optimized inverted pyramid PhC cell over the 850–1200 *nm* wavelength range in Fig. 5(a). The red circles correspond to resonant absorption peaks located at *λ* = 1110, 1130 and 1176 *nm*. Figure 5(b–d) show the in-plane Poynting vector plots over the central *xz*-slice of the inverted pyramid PhC unit cell at these resonances. The energy flow-pattern reveals multiple regions with vortex-like flow and parallel to interface flow of light at these resonances leading to very long dwell-time of photons in the solar cell. On the other hand, Lambertian light trapping assumes that the distribution rays in the cell obeys a probability distribution *f*(*θ*) = 1/*π* cos*θ*, where *θ* is the angle that a ray within the cell makes with the cell-surface normal. According to this distribution, propagation of energy near *θ* = 90° (i.e. parallel to the interface) is insignificant. However, direct solutions of Maxwell’s equations show that a significant amount of energy flows close to *θ* = 90° due to wave-interference based light-trapping in our PhC. Moreover, a ray-optics based picture cannot provide vortices in the power-flow pattern shown in Fig. 5(b–d).

## Electronic Optimization

Collection of the photo-generated carriers, before they recombine, is crucial for high power conversion efficiency in solar cells. Accordingly, the emitter, base and FSF regions of the IBC cell require higher doping levels in order to deflect minority carriers from contacts and other surfaces. However, high doping levels in these regions lead to high Auger recombination. Higher doping also reduces the open-circuit voltage due to larger BGN. Therefore, a careful balance between the peak doping concentration and depths of the Gaussian doping profiles is paramount to exploiting the full potential of wave-interference based light-trapping. For carrier-transport optimizations, we use a 2*D* model of the IBC cell (shown in Fig. 2). The design parameters such as the details of the Gaussian doping profiles and contact widths turn out to be independent of the PhC cell-thickness. For concreteness, we describe in detail the optimization process for a 10 *μm* thick cell.

**Figure 6:**
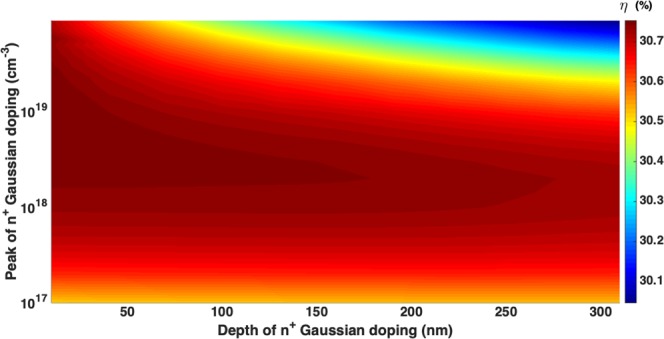
Emitter optimization of 10 *μm*-thick inverted pyramid PhC IBC cell with *N*_*p*0_ = 5 × 10^18^ *cm*^−3^, *σ*_*p*_ = 100 *nm*. Table 2 contains the details of the contact geometry and other simulation parameters. Here, the Gaussian doping profiles are of the form ${N}_{i0}\exp (-\,{z}^{2}/2{\sigma }_{i}^{2})$ for *i* = *n*, *p*. The cell yields a power conversion efficiency of 30.74% at *N*_*n*0_ = 2 × 10^18^ cm^−3^ and *σ*_*n*_ = 220 *nm*.

Figure 6 shows the optimization map for the Gaussian doping profile of the *n*^+^ emitter region. For this optimization, the peak doping concentration (*N*_*p*0_) and doping depth (*σ*_*p*_) of *p*^+^ regions are kept fixed at 5 × 10^18^ *cm*^−3^ and 100 *nm* (corresponding to a total *p*^+^ region depth of 370 *nm*). The specific contact geometry and other parameters used for emitter optimization calculations are given in Table 2. Figure 6 shows that our 10 *μm*-thick IBC cell achieves a conversion efficiency of 30.74% for a peak emitter doping concentration *N*_*n*0_ = 2 × 10^18^ *cm*^−3^ and emitter doping depth *σ*_*n*_ = 220 *nm*, corresponding to a total emitter depth of 760 *nm*. This plot reveals that if we increase *σ*_*n*_ to 300 *nm* (keeping *N*_*n*0_ fixed at 2 × 10^18^ *cm*^−3^ which corresponds to an emitter depth of 1.038 *μm*), the cell efficiency exhibits a negligible drop of 0.01% (additive). Clearly, *N*_*n*0_ = 2 × 10^18^ *cm*^−3^ allows a large tolerance toward emitter-fabrication.

**Table 2:** Parameters used in emitter and base doping optimization of IBC cell as described in Fig. 6.

Parameters	Description
*H*	10 *μm*
*τ*_*SRH*_	10 *ms*
Contact SRV	10 *cm*/*s*
Bulk acceptor doping	5 × 10^15^ *cm*^−3^
*w*_*pcon*_	140 *μm*
*w*_*ncon*_	10 *μm*
*w*_*pn*_	1 *μm*
*w*_*pdop*_	1.1*w*_*pcon*_
*w*_*ndop*_	1.1*w*_*ncon*_

The Auger recombination of the carriers are described by improved Auger model of^11^. The BGN of *Si* and surface recombination at *Si* − *SiO*_2_ interface are modeled according to the details illustrated in the “Methods” section.

**Figure 7:**
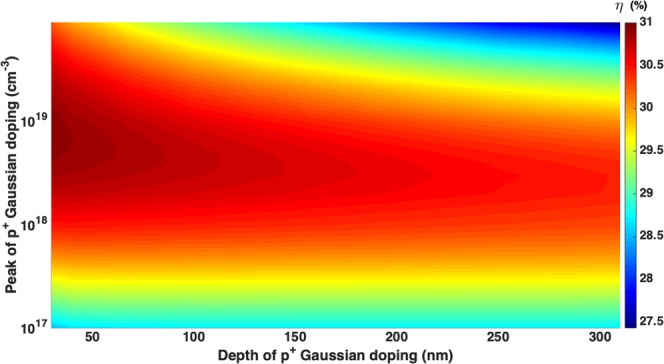
Optimization of *p*^+^ base and FSF regions of the 10 *μm*-thick inverted pyramid photonic crystal IBC solar cell with *N*_*n*0_ = 2 × 10^18^ *cm*^−3^ and *σ*_*n*_ = 220 *nm*. With the contact geometry and other design parameters of Table 2, the choice of *N*_*p*0_ = 4 × 10^18^ *cm*^−3^ and *σ*_*p*_ = 100 *nm* yields 30.75% conversion efficiency.

For the optimization of *p*^+^ regions (shown in Fig. 7), we choose *N*_*n*0_ = 2 × 10^18^ *cm*^−3^ and *σ*_*n*_ = 220 *nm*. For both *p*^+^ base and FSF regions, we assume the same Gaussian doping profile, characterized by peak doping concentration *N*_*p*0_ and depth *σ*_*p*_. Other simulation parameters used in our base doping optimization are given in Table 2. Our cell achieves 30.75% conversion efficiency for *N*_*p*0_ = 4 × 10^18^ *cm*^−3^ and *σ*_*p*_ = 100 *nm*.

The PERC cell described in^4^, has an emitter with *N*_*n*0_ = 3 × 10^18^ *cm*^−3^ and a total depth of 730 *nm*. This is very similar to the optimized emitter of our IBC cell. However, the emitter optimization in^4^ does not involve BGN and is a result of the balance between Auger recombination and sheet resistance. In contrast, the carriers in our IBC cells travel at most 70 *μm* lateral distance, unlike PERC cells where, the electrons inside the emitter region travel several hundreds of microns laterally before they reach front contacts. Consequently, sheet resistance is much less in our IBC cell. In addition to Auger recombination, we have included BGN in the present optimization study. This unavoidable effect limits *N*_*p*0_ and *N*_*n*0_ in a practical cell. The BGN-mediated drop in the open-circuit voltage reduces the power conversion efficiency for large *N*_*p*0_ and *N*_*n*0_.

**Figure 8:**
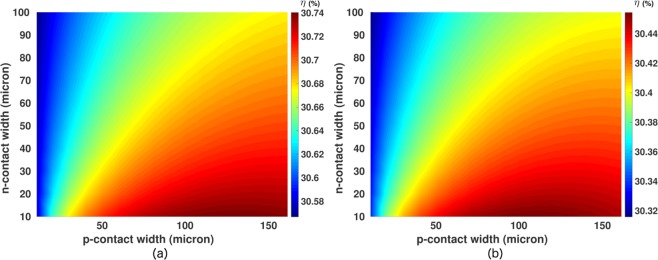
Contact width optimization of inverted pyramid photonic crystal IBC cell with design parameters specified in Table 3. (**a**) For contact SRV = 10 *cm*/*s*, the optimum base and emitter contact widths are 140 *μm* and 10 *μm*, respectively. (**b**) For contact SRV = 100 *cm*/*s*, the optimum width of the base contact is reduced to 110 *μm* and the cell efficiency exhibits a drop of 0.25% (additive).

Figure 8(a,b) show the optimization maps for *p* and *n*-contact widths with contact SRVs 10 *cm*/*s* and 100 *cm*/*s*, respectively. The IBC cell is assumed to be 10 *μm* thick with optimized *p*^+^ and *n*^+^ dopings. Table 3 shows the details of all the simulation parameters used in our contact optimization study. Figure 7(a) reveals that for contact SRV = 10 *cm*/*s* and 10 *μm* emitter-contact width, the optimum value of base contact width, *w*_*pcon*_, is 140 *μm*. As *w*_*pcon*_ increases from 10 *μm* to the optimum value of 140 *μm*, the cell efficiency increases by 0.2% (additive). In comparison to this, the variation of the emitter contact width (*w*_*ncon*_) has even less influence on the power conversion efficiency of the cell. For *w*_*pcon*_ = 140 *μm*, as *w*_*ncon*_ increases from 10 *μm* to 100 *μm*, the power conversion efficiency of the photonic crystal IBC cell drops only by 0.08% (additive). As we increase the contact SRV to 100 *cm*/*s*, Fig. 8(b) shows that the relative influences of the variations in *w*_*pcon*_ and *w*_*ncon*_ on the cell efficiency remain approximately the same as for contact SRV 10 *cm*/*s*. However, the optimum base contact width and maximum power conversion efficiency now have lower values, 110 *μm* and 30.49%, respectively. Although the contact SRV increased by an order of magnitude, the cell efficiency changes only by 0.25% (additive).

**Table 3:** Parameters used in contact width optimization of IBC cell as described in Fig. 8.

Parameters	Description
*H*	10 *μm*
*τ*_*SRH*_	10 *ms*
*w*_*pn*_	1 *μm*
*w*_*pdop*_	1.1*w*_*pcon*_
*w*_*ndop*_	1.1*w*_*ncon*_
Bulk acceptor doping	5 × 10^15^ *cm*^−3^
*N*_*p*0_	4 × 10^18^ *cm*^−3^
*σ*_*p*_	100 *nm*
*N*_*n*0_	2 × 10^18^ *cm*^−3^
*σ*_*n*_	220 *nm*

The Auger recombination of the carriers are described by improved Auger model of^11^. The BGN of *Si* and surface recombination at *Si* − *SiO*_2_ interface are modeled according to the details illustrated in the “Methods” section.

**Figure 9:**
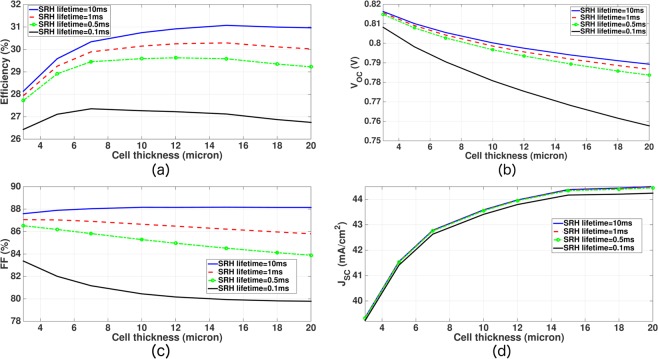
Thickness optimization of thin-silicon inverted pyramid PhC IBC solar cells with optimum lattice constants and dual-layer ARCs, given by Table 1. The cell-design parameters for transport computations are given in Table 4. For *τ*_*SRH*_ = 0.1 and 0.5 *ms*, the optimum IBC cells are 7 and 12 *μm* thick, respectively. For both *τ*_*SRH*_ = 1 *ms* and 10 *ms*, the optimum cell-thickness becomes 15 *μm*.

Using the optimum contact widths and doping profiles obtained above, we now study the optimum thickness of our inverted pyramid PhC IBC cell. A thicker cell leads to a higher short-circuit current due to more light absorption but has a lower open-circuit voltage due to increased bulk recombination of photo-generated carriers. This trade-off leads to an optimum cell-thickness for each choice of *τ*_*SRH*_. The SRH lifetime, determined by bulk defects in the *c*–*Si* wafer, can vary widely depending upon the quality of the fabrication process. A higher SRH lifetime or lower bulk recombination allows larger solar absorption by using thicker *c*–*Si* layer without losing much photo-current in the recombination process. It follows that for a higher SRH lifetime, the optimum cell-thickness is larger and vice versa. In Fig. 9, we consider *τ*_*SRH*_ = 0.1,0.5,1 and 10 *ms* to study the optimum cell-thickness (other simulation parameters appear in Table 4). Figure 9(b–d) show the variation of *V*_*OC*_, *J*_*SC*_ and *FF* with cell-thickness. As the cell-thickness is increased from 3 to 20 *μm*, the *FF* of the IBC cell drops by 4% (additive) for *τ*_*SRH*_ = 0.1 *ms*. As *τ*_*SRH*_ increases, the drop in the *FF* becomes smaller for the same range of cell-thickness variation. For *τ*_*SRH*_ = 10 *ms*, the *FF* becomes almost independent of cell-thickness.

**Table 4:** Parameters used in thickness optimization of inverted pyramid photonic crystal IBC cell as described in Fig. 9.

Parameters	Description
*τ*_*SRH*_	0.1, 0.5, 1 and 10 *ms*
contact srv	10 *cm*/*s*
*w*_*pcon*_	140 *μm*
*w*_*ncon*_	10 *μm*
*w*_*pn*_	1 *μm*
*w*_*pdop*_	1.1*w*_*pcon*_
*w*_*ndop*_	1.1*w*_*ncon*_
bulk acceptor doping	5 × 10^15^ *cm*^−3^
*N*_*p*0_	4 × 10^18^ *cm*^−3^
*σ*_*p*_	100 *nm*
*N*_*n*0_	2 × 10^18^ *cm*^−3^
*σ*_*n*_	220 *nm*

The Auger recombination of the carriers are described by improved Auger model of^11^. The BGN of *Si* and surface recombination at *Si* − *SiO*_2_ interface are modeled according to the details illustrated in the “Methods” section.

Figure 9(a) shows the variation of power conversion efficiency of our IBC cell with cell-thickness for various choices of *τ*_*SRH*_. For *τ*_*SRH*_ = 0.1 *ms* and 0.5 *ms*, the optimum IBC cells are 7 *μm* and 12 *μm* thick with conversion efficiencies 27.35% and 29.63%, respectively. For both *τ*_*SRH*_ = 1 *ms* and 10 *ms*, the optimum cell-thickness becomes 15 *μm* with power conversion efficiencies 30.29% and 31.07%, respectively.

**Figure 10:**
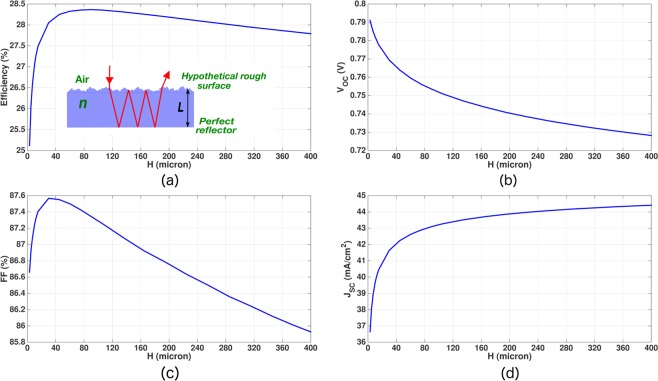
Optimization of solar cells with ray-optics based Lambertian light-trapping. The cells are assumed to have same doping profiles as our inverted pyramid PhC IBC solar cell (given by Table 4). The Lambertian cells are assumed to have contact SRV = 10 *cm*/*s* and *τ*_*SRH*_ = 10 *ms*. The Auger recombination is modeled using the improved Auger model of^11^. For BGN, we use the model illustrated in the “Methods” section. In comparison to a lossless, undoped Lambertian cell with maximum theoretical efficiency of 29.43% and optimum thickness 110 *μm*^10^, inclusion of practical doping profiles, bulk recombination and surface recombination reduces the maximum theoretical efficiency of the Lambertian cell to 28.37% with an optimum thickness of 90 *μm*. In contrast, our inverted pyramid PhC IBC solar cell with same design parameters achieves 31.07% conversion efficiency with an optimum thickness of 15 *μm*.

It is instructive to compare the optimization of our realistic IBC cell with that of a hypothetical, ideal Lambertian cell. Here, we include the same surface recombination mechanism and SRH lifetime as used in our best IBC cell. The hypothetical Lambertian cell is also endowed with the same doping levels as the *p*-type bulk, *n*^+^ and *p*^+^ regions of our IBC cell. The optimum thickness of the Lambertian cell is found to be 90 *μm* with a maximum conversion efficiency of 28.37% (shown in Fig. 10). Thus, our thin-*Si* photonic crystal solar cell offers 2.7% (additive) higher conversion efficiency than the limiting efficiency of a Lambertian cell with practical doping configurations and loss mechanisms. Table 5 compares the performance of our inverted pyramid PhC IBC solar cell with the hypothetical Lambertian solar cell.

**Table 5:** Comparison of our inverted pyramid PhC IBC solar cells with the hypothetical Lambertian solar cell at 25 °*C*.

Cell type/light trapping and transport model	Cell-thickness (*μm*)	Bulk recombination model	Surface recombination	*V*_*OC*_(*V*)	*J*_*SC*_(*mA*/*cm*^2^)	*FF*(%)	*η*(%)
Hypothetical Lambertian, undoped	110	Improved Auger^11^, *τ*_*SRH*_ = ∞	SRV = 0	0.7613	43.31	89.26	29.43
Hypothetical Lambertian, doping profiles in Table 4	90	Improved Auger^11^, *τ*_*SRH*_ = 10 *ms*	Contact SRVs 10 *cm*/*s*	0.7535	43.10	87.34	28.37
Inverted pyramid PhC, 2*D* transport							
(design parameters in Table 4)	15	Improved Auger^11^, *τ*_*SRH*_ = 10 *ms*	Contact SRVs 10 *cm*/*s*	0.7940	44.39	88.17	31.07
Inverted pyramid PhC, 2*D* transport							
(design parameters in Table 4)	15	Improved Auger^11^, *τ*_*SRH*_ = 10 *ms*	Contact SRVs 100 *cm*/*s*	0.7908	44.39	87.67	30.77

The inverted pyramid PhC cells employ wave-interference based light trapping. The design parameters of the PhC IBC solar cells are given in Table 4. All cells include band gap narrowing and optical absorption throughout the 300–1200 *nm* wavelength range.

Lifetime measurements of well-passivated *c*–*Si* samples (Fig. 5 in^11^) have shown that the effective lifetime (*τ*_*eff*_) of *p*-type samples with a bulk doping concentration of 5 × 10^15^ *cm*^−3^ is approximately 10 *ms*. Since, *τ*_*SRH*_ > *τ*_*eff*_ (as a result of the relation: *τ*_*eff*_ = (1/*τ*_*SRH*_ + 1/*τ*_*Aug*_)^−1^), *τ*_*SRH*_ = 10 *ms* is practically attainable. We now consider our 15 *μm*-thick IBC PhC cell, with optimized light-trapping, to study the degradation of performance with lower quality electronic parameters. We delineate below, how higher SRH lifetime, poor contact quality and non-optimized FSF, lower the efficiency of our PhC IBC cell.

**Figure 11:**
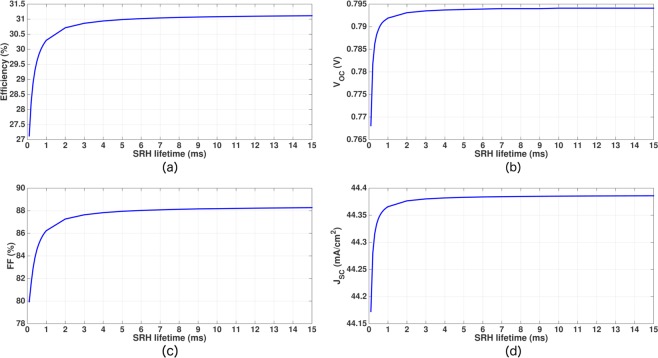
Effect of SRH lifetime variation on the performance of the 15 *μm*-thick inverted pyramid PhC IBC solar cell with the other design parameters given in Table 4. The power conversion efficiency increases rapidly as *τ*_*SRH*_ increases from 0.1 *ms* to 1 *ms*.

The effect of higher *τ*_*SRH*_ is shown in Fig. 11. Other simulation parameters are same as given in Table 4. As shown in Fig. 11(d), *J*_*SC*_ exhibits little variation with *τ*_*SRH*_ (only ~0.22 *mA*/*cm*^2^ over the entire range of 0.1 *ms* ≤ *τ*_*SRH*_ ≤ 15 *ms*). In contrast, *V*_*OC*_ and *FF* of the cell increase significantly as *τ*_*SRH*_ changes from 0.1 *ms* to 1 *ms*. Within this range of *τ*_*SRH*_, *V*_*OC*_ increases from 768 *mV* to 792 *mV* and *FF* increases from 79.95% to 86.24%. For *τ*_*SRH*_ > 3 *ms*, *V*_*OC*_ of our IBC cell falls very close to its saturation value of 794.1 *mV*. Similarly, *FF* of the cell almost reaches its saturation value of 88.2% as *τ*_*SRH*_ becomes larger than 5 *ms*. Overall conversion efficiency of our 15 *μm*-thick IBC cell increases steeply from 27.12% at *τ*_*SRH*_ = 0.1 *ms* to 30.3% at *τ*_*SRH*_ = 1 *ms*. Power conversion efficiency of our cell crosses the 31% threshold for *τ*_*SRH*_ > 5 *ms*. Clearly, *τ*_*SRH*_ > 1 *ms* is a prerequisite for photonic crystal IBC cells to achieve efficiency beyond 30%.

**Figure 12:**
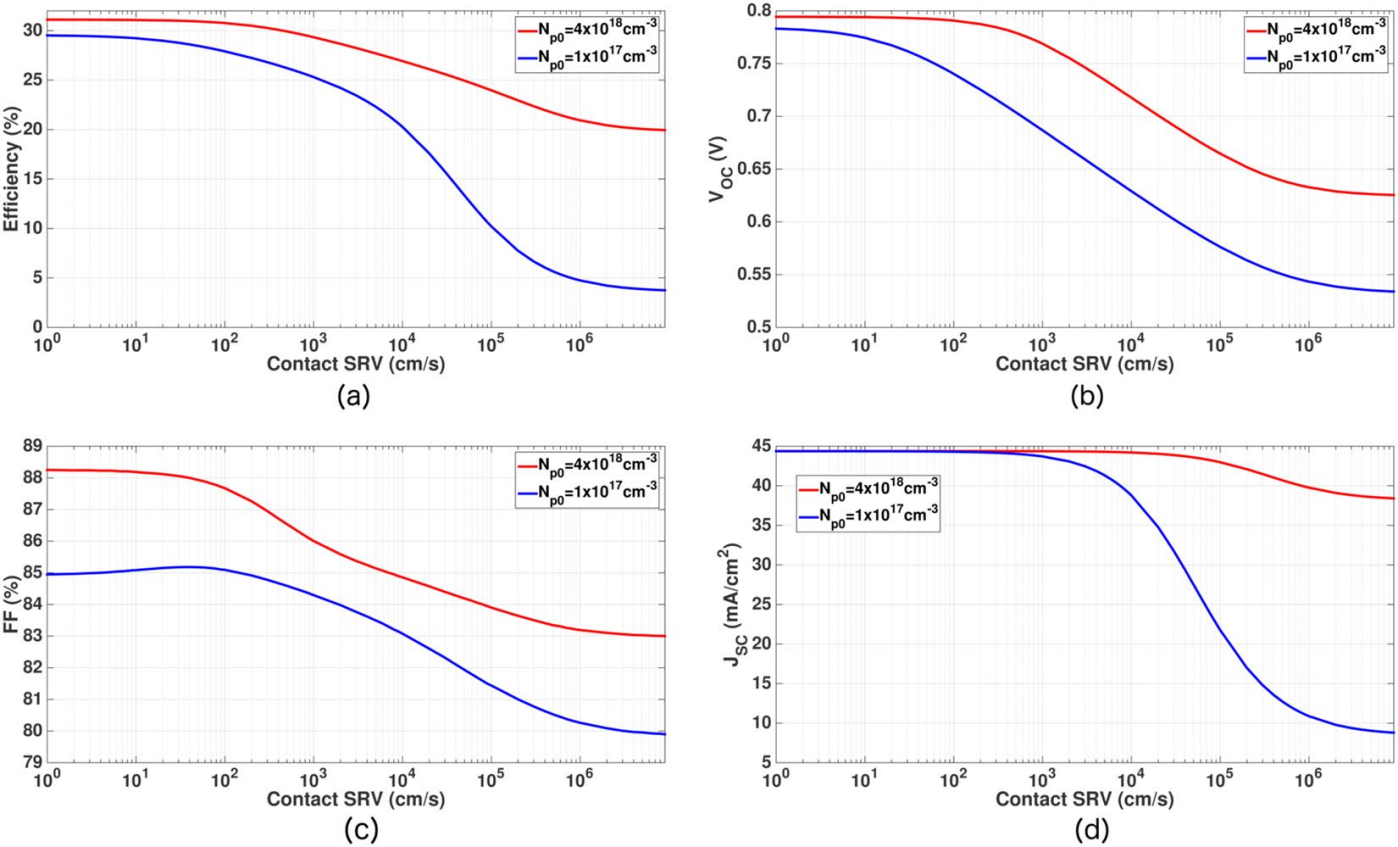
Effect of contact SRV on the performance parameters of the proposed inverted pyramid PhC IBC cell with *H* = 15 *μm* and *τ*_*SRH*_ = 10 *ms* (other design parameters are specified in Table 4). The red curve corresponds to optimum FSF and BSF doping, showing a more gradual drop in the cell efficiency as contact SRV increases. In contrast, a rapid degradation in cell efficiency (blue curve) when FSF/BSF dopings are improperly chosen.

We now consider the effect of increased contact SRV and non-optimized FSF/BSF on our 15 *μm*-thick photonic crystal IBC cell. Apart from *τ*_*SRH*_ = 10 *ms* and a variable contact SRV, all other simulation parameters are given by Table 4. Figure 12(a) shows that the power conversion efficiency of our IBC cell with optimized FSF and BSF (i.e. *N*_*p*0_ = 4 × 10^18^ *cm*^−3^ and *σ*_*p*_ = 100 *nm*) undergoes only 0.3% (additive) drop leading to 30.77% efficiency when the contact SRV is increased from 10 *cm*/*s* to 100 *cm*/*s* (red curves in Fig. 12). In contrast, the blue curve in Fig. 12(a) shows a 5% (additive) drop in the conversion efficiency for the same change in contact SRV, in a cell with inadequate FSF and BSF (*N*_*p*0_ = 1 × 10^17^ *cm*^−3^ and *σ*_*p*_ = 100 *nm* in this particular example). When the contact SRVs are extremely high (~10^6^ *cm*/*s*), the IBC cell with optimum FSF/BSF doping retains ~20% power conversion efficiency. This is in sharp contrast to the cell with inadequate FSF/BSF where the conversion efficiency drops to ~5%.

## Conclusions

Through detailed and precise design optimization, we have identified a route to 31% power conversion efficiency in thin-film crystalline silicon solar cells. The architecture consists of a flexible 15 *μm*-thick *c*–*Si* sheet patterned as a square-lattice, inverted micro-pyramid photonic crystal with a grid of interdigitated back contacts. By choosing the micro-pyramid lattice spacing comparable to the wavelength of near-infrared light, it is possible to achieve remarkable wave-interference-based light-trapping throughout the 800–1200 *nm* wavelength range. Together with an optimized anti-reflection coating, this leads to overall solar absorption in the 300–1200 *nm* range, well above the so called Lambertian limit. This unprecedented amount of light absorption in a thin-film, indirect band gap semiconductor, exploits the wave nature of light and suggests a paradigm shift in solar cell design. It leads to the remarkable conclusion that thin, flexible, silicon solar cells may outperform their traditional, thick, inflexible counterparts. Given the advanced technologies available for silicon surface passivation, it suggests that thin-film silicon may provide higher power conversion efficiency than any other single material of any thickness.

Our predictions remain robust over a reasonable range of photonic crystal structure parameters as well as a viable range of surface recombination velocities at the silicon-contact interfaces. The vital role of front and back surface fields obtained by realistic doping profiles was delineated. Major deviations from the prescribed profiles were shown to cause rapid degradation of solar cell performance. By elucidating the optimized photonic and electronic architecture, together with deviations from the optimum parameter choices, we provide a detailed roadmap for experimental efforts to realize power conversion efficiency beyond 30% in a thin-silicon solar cell.

## Methods

### Long wavelength absorption in crystalline silicon

Sub-gap absorption in *c*–*Si* arises from two distinct mechanisms. The first is electronic bandgap narrowing (BGN), denoted by Δ*E*_*g*_ (in *eV*). This allows *c*–*Si* to absorb sub-gap photons with energies less than *E*_*g*_ but above (*E*_*g*_ − Δ*E*_*g*_), where *E*_*g*_ is the bandgap energy (in eV) of unperturbed *c*–*Si*. In Sentaurus, Δ*E*_*g*_ is estimated using Schenk’s model^40^ and leads to a slight drop in *V*_*OC*_. The second mechanism allows *c*–*Si* to absorb photons with energies less than (*E*_*g*_ − Δ*E*_*g*_) and originates from the exponentially decaying Urbach tail below the continuum band edge^41,42^. For non-crystalline solids, static disorder contributes to an exponential band tail of localized states below the electronic band edge. In *c*–*Si*, a similar tail of phonon-assisted optical absorption gives rise to mobile electron-hole pairs^43,44^. The sub-gap absorption is characterized by an exponential of the form: *α*(*ν*) ~ *exp*[{*hν* − *E*_*G*_(*T*)/*E*_0_(*T*)}], where *ν* is the optical frequency, *E*_*G*_(*T*) is the downshift of the continuum band edge corresponding to BGN and *E*_0_(*T*) is the Urbach slope.

**Figure 13:**
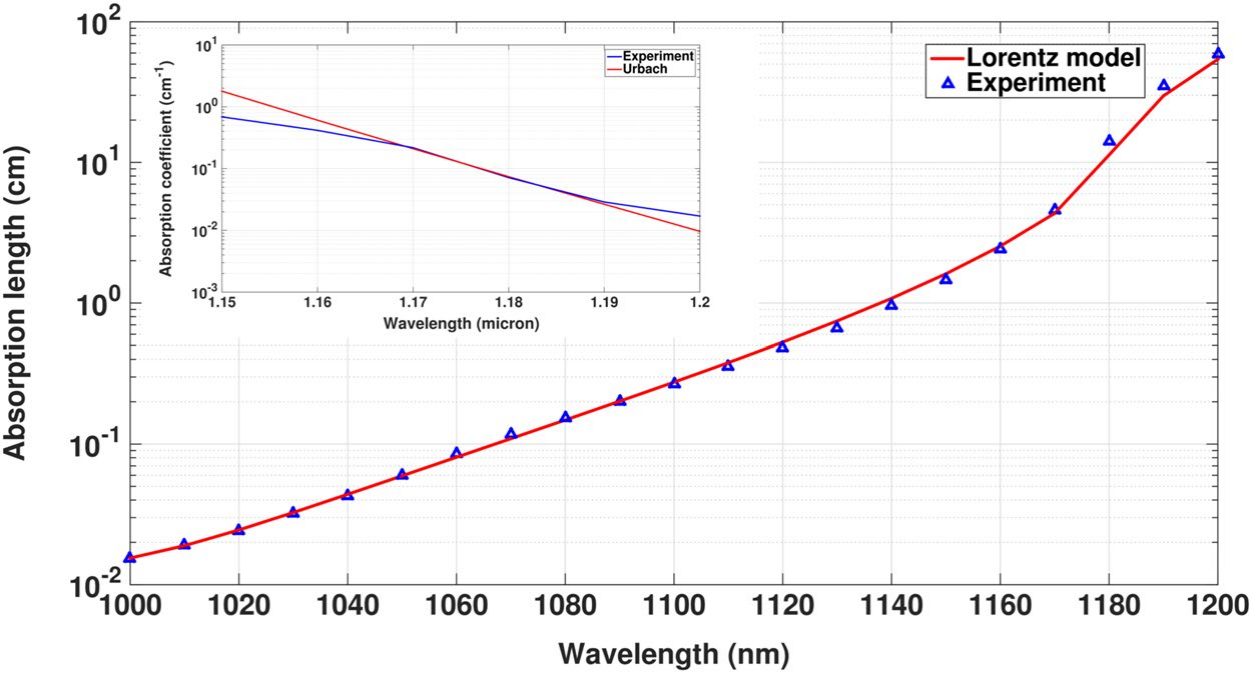
Fitting of absorption length of *c*–*Si* in 1000–1200 *nm* wavelength range with experimental data obtained from^52^. Fitting parameters are given in Table 6. Inset: Urbach slope exhibited by the experimental absorption coefficient.

In order to model the sub-gap absorption, we take the frequency dependent dielectric constant of *Si* over the 1000–1200 *nm* wavelength range from^52^ and fit it to a sum of Lorentz oscillator terms: $\varepsilon (\omega )={\varepsilon }_{\infty }+{\sum }_{j}\frac{{\rm{\Delta }}{\varepsilon }_{j}{\omega }_{pj}^{2}}{({\omega }_{pj}^{2}-2i\omega {\gamma }_{j}-{\omega }^{2})}$. The fitting parameters *ε*_∞_, *ω*_*pj*_, Δ*ε*_*j*_ and *γ*_*j*_, given in Table 6, are obtained using an open MATLAB program^53^. We compare the absorption length *λ*/4*πk* (where *k* is the imaginary part of the refractive index) calculated form our fit and that obtained from^52^ in Fig. 13. The measured value of Urbach slope of *c*–*Si* at 300*K* is 8.5 ± 1.0 *meV*^54^. In comparison to this experimental data, microscopic modeling of the optical-absorption edge due to acoustic and optical phonons yields a slope of 8.6 *meV*^43,44^. The inset shows that the experimental data from^52^ exhibits an Urbach slope of 8.6 *meV* over the 1160–1190 *nm* wavelength range.

**Table 6:** Fitting parameters for experimental *Si* dispersion data of^52^.

Wavelength range	${\epsilon }_{\infty }$	Δ*ε*_*j*_	*ω*_*pj*_(×10^3^ *μm*^−1^)	*γ*_*j*_(×10^3^ *μm*^−1^)
1000–1200 *nm*	1.0	0.971156	0.001805	0.000000
		7.244785	0.006785	0.000001
		0.000580	0.001018	0.000047
		2.519084	0.002291	0.000000
		−0.057262	0.001237	0.000004

### Surface recombination at insulator-silicon interface

For all our transport calculations, we use SRH statistics for the insulator-*Si* interface. According to this model, the recombination rate at the *Si*-insulator interface is given by^46^:

2 $${R}_{surface}^{SRH}=\frac{({n}_{s}{p}_{s}-{n}_{i}^{2})}{({n}_{s}+{n}_{i})/{S}_{p0}+({p}_{s}+{n}_{i})/{S}_{n0}}$$

where, *S*_*j*0_ = *v*_*th*,*j*_*σ*_*j*_*D*_*interface*_ with *j* = *n*,*p* (*v*_*th*,*j*_ is the thermal velocity, *σ*_*j*_ is the capture cross-section, *D*_*interface*_ is the interface trap density at the oxide-semiconductor interface), *n*_*s*_ and *p*_*s*_ are electron and hole concentration at the *Si* surface and ${n}_{i}=\sqrt{{N}_{e}{N}_{h}}\exp (-\,{E}_{g}(T)/2{k}_{B}T)$. Here, *T* is the temperature (in *K*) and *E*_*g*_(*T*) denotes the bandgap of *Si*. *N*_*e*_ and *N*_*h*_ are defined in terms of the electron/hole effective mass ${m}_{e}^{\ast }/{m}_{h}^{\ast }$ and Planck’s constant *h* as: ${N}_{j}=2{(\frac{2\pi {m}_{j}^{\ast }{K}_{B}T}{{h}^{2}})}^{3/2}$ with *j* = *e* and *h* for electrons and holes, respectively. For electrons, ${v}_{th}=\sqrt{\frac{3KT}{{m}_{e}^{\ast }}}=1.12\times {10}^{7}cm/s$ for ${m}_{e}^{\ast }=1.08{m}_{e}$ and *T* = 298*K*. The thermal velocity of holes is slightly lower due to higher effective mass $(\, \sim \,1.5{m}_{e}^{\ast })$. We set *D*_*interface*_ = 3 × 10^9^ *cm*^−2^ according to the measured value of the near-midgap trap density at the *Si*-insulator interface in^47^. We take *σ*_*p*_ = 6 × 10^−17^ *cm*^2^ for these traps from the measured data on capture cross-sections (Fig. 6 in^48^). This figure also shows that the measured value of *σ*_*n*_ varies over a large range. The choice of *σ*_*n*_ = 6 × 10^−16^ *cm*^2^ results in a *S*_*n*0_ that closely approximates the effective SRV of the state of the art measurements in^11^. Accordingly, we choose *S*_*n*0_ ≈ 20.16 *cm*/*s* and *S*_*p*0_ ≈ 1.7 *cm*/*s* for all our transport calculations.
